# Veteran participation in the *All of Us* Research Program: applying an intersectionality lens to evaluate participant diversity

**DOI:** 10.1186/s12874-025-02588-0

**Published:** 2025-05-24

**Authors:** Lauren O. Thomann, Katherine A. Hanson, Jane Plomp, Jason L. Vassy, Sherilyn J. Sawyer

**Affiliations:** 1 VA Palo Alto Healthcare System, Palo Alto, CA U.S.; 2 Boston VA Research Institute, VA Boston Healthcare System, Boston, MA U.S.; 3 VA Boston Healthcare System, Harvard Medical School, Mass General Brigham, Boston, MA U.S.

**Keywords:** Veteran, Intersectionality, Engagement approaches, Representation, Diversity, Identity, Biomedical research, *All of Us* Research Program, VA

## Abstract

**Background:**

Many segments of the population are underrepresented in biomedical research (UBR). Lack of diversity in health research limits understanding of individual and population level differences and risks the generalizability of study results. Examining intersectionality among VA-enrolled Veteran participants in the *All of Us* Research Program offers a first look at in-depth understanding of Veteran identity.

**Methods:**

Multi-modal approaches to engagement and recruitment were utilized to enroll a diverse cohort of Veterans nationally from 2018 to 2024. *All of Us* data were analyzed across eight UBR categories and their intersections to highlight the complexity of Veteran identity.

**Results:**

Veteran *All of Us* participants reflect the diversity of Veterans nationwide. *All of Us* participant metrics shed new light on the diversity of the Veteran population, with over 75 unique UBR combinations identified among participants, and over 90% of participants meeting the criteria for at least one UBR category.

**Conclusions:**

The use of a broad spectrum of engagement approaches was shown to be successful for reaching a more diverse Veteran base, and complex intersectional identities among Veterans are described. Greater understanding of intersectionality and its significance to representation can bolster the adaptation of Veteran engagement methodology in research and broader healthcare settings.

## Background

Lack of diversity in health research limits understanding of individual and population level differences in disease progression and medication response and risks the generalizability of study results [1–3]. As a growing body of literature describes, it is increasingly understood that many segments of the population are underrepresented in biomedical research (UBR), including non-White racial and ethnic groups, women, older individuals, people living in rural areas, those with lower socioeconomic status (SES), and individuals with disabilities [4–6]. This is problematic because underrepresented groups experience health and healthcare differently; for example, people of color are less likely to receive adequate mental health care and experience higher rates of being uninsured, individuals with lower incomes are more likely to experience delays in care, and women experience periods of “poor health” at a higher rate than men and lower treatment efficacy [7–9]. Along this vein, reduced access to research thus limits the health benefits received from new insights [10]. Moreover, Hindorff et al. [11] summarize that many recent research breakthroughs themselves have been limited by a lack of diversity. Specifically, Popejoy and Fullerton [3] report on novel genomic findings, but note limitations on generalizability and disease risk prediction due to majority sampling from ancestrally homogeneous groups. Velicer et al. [12] further describe the challenge of developing widely effective interventions without having sufficient representation from demographic subgroups in research, noting the significance of recruitment methodology focused on diverse enrollment.

Consideration of intersectionality, which helps to explain how multidimensional identities and social characteristics interact to influence health opportunities and outcomes, can be particularly valuable when trying to achieve diverse representation among research participants and equity in biomedical research advancements and medical innovation [13–15]. Intersectionality was first discussed in the literature by Kimberle Crenshaw to highlight the marginalization of Black women in society, demonstrating how their unique experiences of race *and* sex discrimination have been largely overlooked [16], and has since been used to more broadly express the importance of considering how different groups and populations are impacted by complex identity and experiences [15]. Specific to United States Military Veterans, Blevins and Blevins [17] describe using an intersectional lens to emphasize the need to center Veteran policy and program practices on the “multiply marginalized.” Use of research engagement practices taking intersectionality into account can help achieve greater diversity and minimize oversimplification of population characteristics [15]. Additionally, by viewing participant characteristics through an intersectionality lens, researchers can begin to overcome historically inaccurate associations between biology, complex social structures, and lived experience [13, 14].

In order to achieve equitable access to research and diverse research participation, barriers to participation must be mitigated. Aside from well documented logistical barriers such as high demand on time and distance to research facilities [18, 19], additional obstacles that commonly affect underserved populations include restrictive eligibility criteria, steep costs to participate, limited health insurance coverage, and distrust of the healthcare system [6, 11]. Understanding unique identities and accompanying barriers to research participation facilitates development of more equitable research opportunities, enabling greater cohort diversity and increasing the generalizability of research findings.

The *All of Us* Research Program is one of numerous efforts by the National Institutes of Health (NIH) focused on increasing inclusion of individuals and groups historically underrepresented in biomedical research, in response to legislation passed over the last two decades to better address health disparities [20, 21]. To date, over 848,000 U.S. residents have enrolled, with more than 80% of participants representing one or more UBR categories [22]. With its size, diversity, and wide-ranging capture of datapoints, the *All of Us* database is already being broadly used by researchers, lending meaningful insights into how a range of genetic and environmental factors along with lived experience intersect to influence overall health [23–25].

United States Military Veterans are a substantial, marginalized, and complex UBR population disproportionately affected by preventable health outcomes and often distrustful of government institutions [26, 27]. However, Veteran diversity is not well characterized beyond general demographics such as age, sex, and race/ethnicity [28]. Focusing on basic demographics alone cannot provide understanding of the full spectrum of identity and experience, potentially contributing to health inequities [29]. Although major Department of Veterans Affairs (VA) research studies have enrolled diverse Veterans nationally, including the Million Veteran Program, which cites having over 25% Veteran enrollees from minority racial backgrounds and over 10% women Veteran enrollees [30], *All of Us* sought to expand diversity further. The VA partnered with the NIH at the onset of the *All of Us* Research Program in 2017 to leverage VA’s national healthcare system to ensure Veteran representation in *All of Us* research.

Recruitment and engagement approaches were continuously revised and expanded upon to enroll diverse Veterans in *All of Us* at 18 engaged VA medical center systems, event venues nationally, and via remote means from 2018 to 2024. Here we (1) describe our methods to date for inclusive engagement of Veterans in *All of Us*, (2) present the intersectional identities of enrolled Veterans, and (3) share initial insight into the complex identity of Veterans using an intersectional approach to inform future research methodologies and support more equitable access to research opportunities, healthcare, and social services.

## Methods

*All of Us* Research Program participation consists of digital consent, completion of a series of health and lifestyle surveys, sharing electronic health records (EHRs), and providing physical measurements and bio-samples [20]. The program uses a decentralized platform and is primarily digital in nature, so full participation can be completed remotely and independently. To ensure program enrollment offerings are widely available, in-person support and specimen collection are also offered at participating clinics throughout the United States. Participant-provided data from surveys and EHRs as well as genetic data from bio-samples are compiled in a data repository and accessible to qualified and registered researchers through the Researcher Workbench [22]. The data available in the workbench are from participants reflective of the diversity of the United States, but do not constitute a representative sample, as the program is open to all interested participants and recruitment is not based on probability sampling [31].

Eighteen VA healthcare systems across 16 states plus Puerto Rico enrolled Veterans into *All of Us*, with active direction from a national coordinating center. Enrollment sites within each healthcare system were located in large medical centers in urban and suburban areas and also within community-based outpatient clinics (CBOCs) to increase ease of access for Veterans. Invitation mailings were sent to Veterans within 50 miles of each enrolling VA medical center, providing program information, a prepaid response postcard, instructions for online enrollment, and contact information for a Veteran-dedicated *All of Us* support center.

A range of pilot outreach approaches and community engagement tools were applied to broaden program reach and overcome barriers to participation (refer to Table 1). Efforts included special enrollment events in locations not served by an enrolling VA medical center (e.g., Wyoming, Tennessee), a remote engagement campaign consisting of invitation mailings focused on driving enrollment of younger Veterans at home including provision of a bio-sample by U.S. mail, and the building of key connections with community hubs and Veteran Service Organizations (e.g., American Legion, Veterans of Foreign Wars) to increase awareness and provide program opportunities in Veteran-familiar settings. Activation of CBOCs, use of a “Bring Your Own Kit” (BYOK) effort, which involved sending bio-sample kits directly to participant homes, as well as previously noted special enrollment events were implemented to increase rural-living Veteran participation. Partnerships with Veteran participant champions were fostered to expand program reach and build trust with marginalized Veteran groups, including women Veterans, Veterans of color, Veterans who identify as LGBTQ+, and Veterans with disabilities, with support from notable networks like the Women Marines Association, National Association of Black Military Women, VHA LGBTQ+ Health Program, and Paralyzed Veterans of America.

**Table 1:** Approaches implemented to meet key engagement challenges

Engagement Challenge	Implemented Approach
Simplify enrollment logistics for regular users of VA healthcare in 16 states and Puerto Rico	Engage Veterans in VA medical centers and VA community-based outpatient clinics (CBOCs) and assist with enrollment on-site scheduling adjacent to health-related visits
Increase program geographic reach by engaging Veterans in locations lacking an enrolling VA medical center, providing in-person enrollment support	Special enrollment events within Veteran community settings in rural and suburban locations not served by *All of Us*-engaged VA medical centers
Overcome barriers to participation for Veterans able to self-navigate digital enrollment, such as travel time, scheduling constraints, geography, and mobility limitations to increase representation of younger Veterans	Remote engagement campaign offering participation from home via *All of Us* participant portal including providing saliva by mail
Engage interested Veterans who do not often utilize VA medical center services, increasing participation of those who may be inconvenienced by medical center research appointment	Partnerships with Veteran Service Organizations and other key community groups to provide convenient engagement and enrollment opportunities outside of the medical center setting, meeting Veterans where they are
Increase program visibility and awareness via familiar and trusted sources outside the medical center	Advertising and engagement including billboards, newspaper ads and interviews, radio ads, and podcasts featuring diverse and/or local Veterans and researchers
Expand rural-living Veteran representation by offering a way to provide program bio-samples collected in their local VA blood lab when they visit for routine health care	Bring Your Own Kit (BYOK) - a hybrid enrollment experience with at-home participant mediated enrollment coupled with in-VA bio-sample donation
Overcome lack of program familiarity and trust within communities of Veterans from minority backgrounds and encourage participation	Forge relationships with notable participant champions and ambassadors to share their program experience, amplifying program messaging and increasing trust

A range of strategies were employed to expand engagement to Veterans nationally based on highlighted challenges

Data from 27,083 Veteran participants who completed consents and an initial survey on demographics, education, work, and home life were compiled by the *All of Us* Data and Research Center (DRC). The DRC analyzed survey responses to report participant demographics and to track program goals for enrolling populations previously underrepresented in biomedical research. UBR categories (refer to Table 2) were previously defined and prioritized by *All of Us* leadership after extensive literature review and interest-holder consultation, and include age, race/ethnicity, disability, income, educational attainment, geography, sexual and gender minority (SGM), and healthcare access and utilization (HAU) [6]. The DRC provided counts of VA-enrolled Veteran participants by the UBR categories for which they met the criteria.

**Table 2:** *All of Us* UBR groups and associated characteristics

UBR Category	Characteristics
Age	Individuals under 18 years old or older than 65 years old, as determined by date of birth provided by participant. (Of note, this effort did not include enrollment of any individuals under the age of 18.)
Disability	Individuals self-identifying with a physical or cognitive disability based on a series of survey questions
Education	Individuals without a high school diploma or equivalent based on participant response to educational attainment question
Geography	Individuals who reside in rural and non-metropolitan areas, as indicated by participant**-**provided zip code
Healthcare Access and Utilization (HAU)	Individuals who have not had a needed medical visit in the past 12 months or cannot readily obtain or pay for needed care as reported in survey response
Income	Individuals reporting household incomes equal to or below 200% of the Federal Poverty Level
Race/Ethnicity	Individuals who self-identify as other than White and non-Hispanic (e.g., Asian; Hispanic, Latino, or Spanish; etc.)
Sexual and Gender Minority (SGM)	Individuals who self-report as intersex, or identify as any sexual orientation other than straight (e.g., gay; bisexual; asexual; etc.), and/or who identify as any gender other than man or woman (e.g., transgender; non-binary; etc.)

The characteristics of UBR groups, as identified by the *All of Us* Research Program, are outlined above [6]. A participant’s belonging to any of these UBR groups is determined by their survey responses. Full surveys are available via the Survey Explorer in the *All of Us* Research Hub [22]

National Veteran demographics were obtained from the Veteran Population Project Model developed by the National Center for Veterans Analysis and Statistics and reflect the best available data for the 18.3 million living Veterans as of September 30, 2023 [28]. Basic demographics of VA *All of Us* participants were collected from participant-provided information reported in *All of Us* operational data. Note that the National Center for Veterans Analysis and Statistics and *All of Us* report Veteran race/ethnicity differently, resulting in slightly different categorizations for Veterans nationally and for Veteran *All of Us* participants. *All of Us* participants may report race/ethnicity by selecting any/all of the following categories: American Indian or Alaska Native; Asian; Black, African American, or African; Hispanic, Latino, or Spanish (HLS); Middle Eastern or North African; Native Hawaiian or other Pacific Islander; White. These categories were collapsed to mirror the national data as closely as possible.

All data were analyzed using SAS software, version 9.4 (SAS Institute, Cary, NC) and R, version 4.3.0. In accordance with *All of Us* publication policy, groups containing fewer than 20 participants were removed from reported figures to protect participant privacy. Figures for publication were produced using BioRender.

## Results

Using outreach and enrollment approaches reported in Table 1, we engaged Veterans in all 50 States, plus the District of Columbia, Puerto Rico, and the U.S. Virgin Islands. Approaches were implemented to provide program engagement and enrollment opportunities for a variety of Veteran communities with the aim of increasing enrollee representation. Through combined use of these approaches, 27,083 Veterans enrolled in *All of Us* with the VA and completed an initial survey between March 2018 and October 2024.

**Fig. 1:**
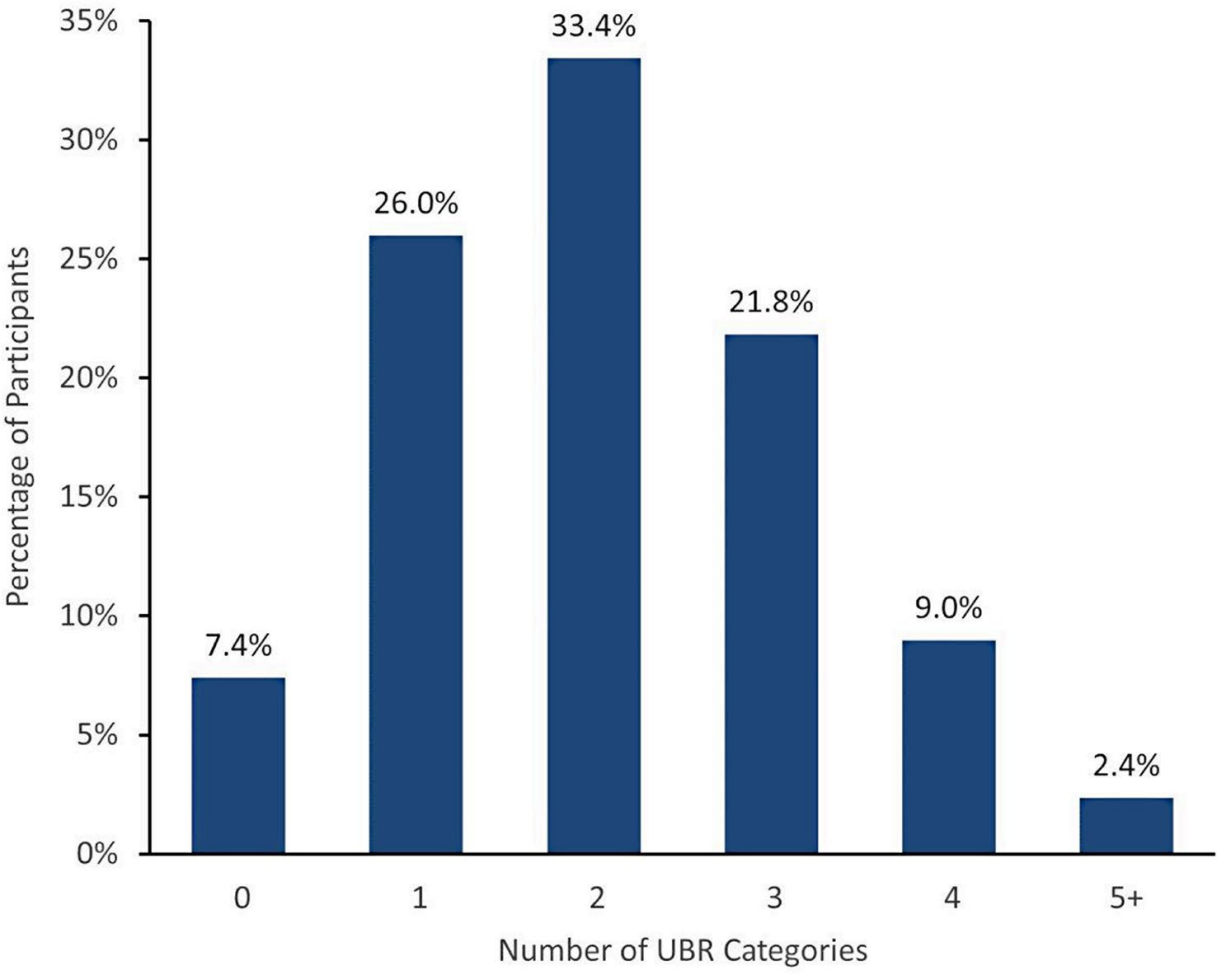
Participant Categories Underrepresented in Biomedical Research (UBR). Percentage of VA participants (*N* = 27,083) identified as belonging to 0–5 + categories considered underrepresented in biomedical research

**Fig. 2:**
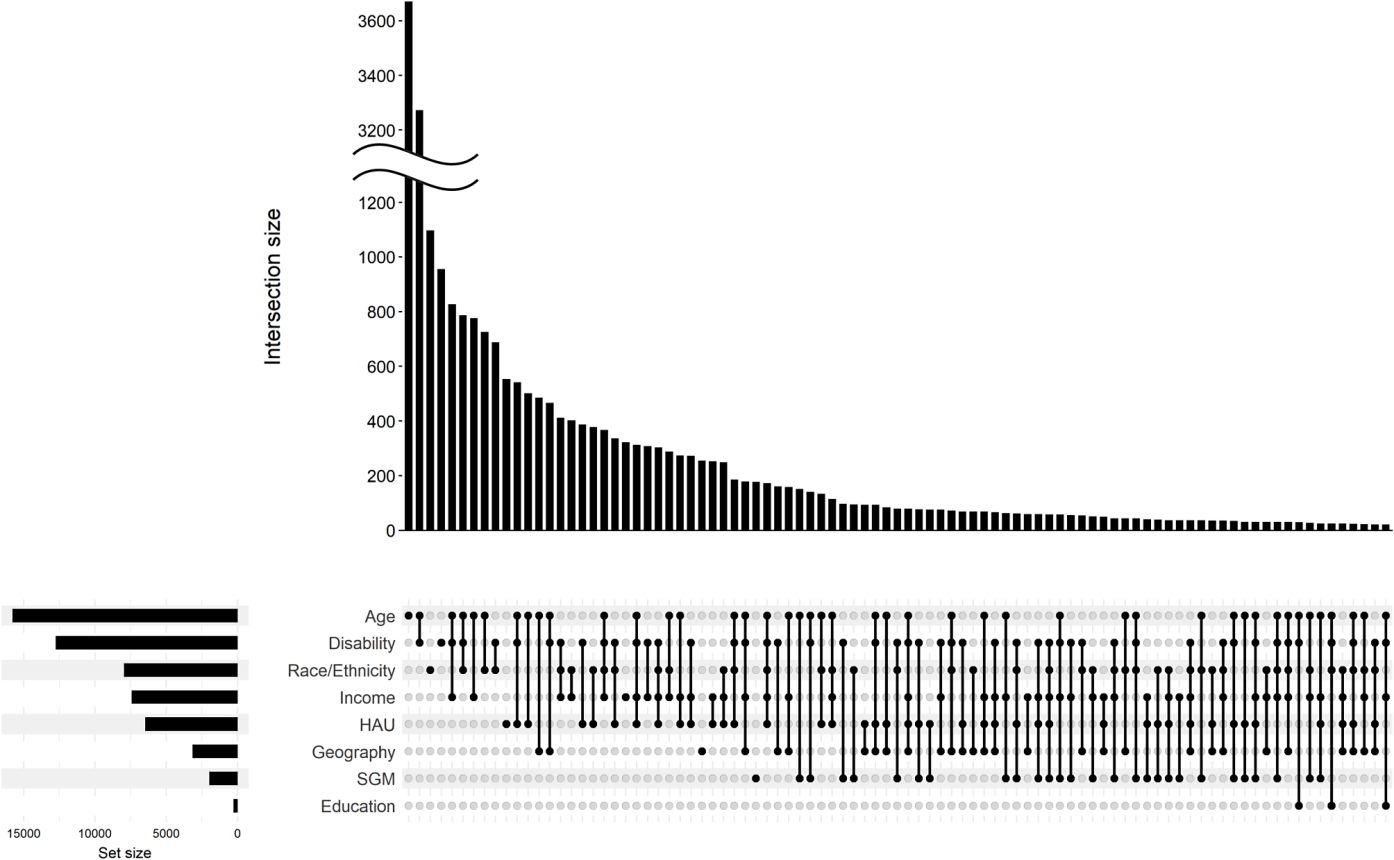
Intersectionality of VA *All of Us* Participants. Intersecting UBR categories for VA *All of Us* participants were analyzed and displayed via UpSet plot (M. Krassowski, 10.5281/zenodo.3700590). Participants who do not meet the criteria for any UBR category are excluded from this analysis. SGM = sexual and gender minority; HAU = healthcare access and utilization Details of each intersection are displayed by a dot and line grid, while the size of the intersection (number of participants in the intersection) is displayed in the bar graph. Set Size is indicative of the total count of every Veteran in the UBR category, either alone, or in combination with additional categories. Figure reflects 25,078 Veterans overall with set size by UBR category as follows: Age (*N* = 15,762); Disability (*N* = 12,764); Race/Ethnicity (*N* = 7,957); Income (*N* = 7,432); HAU (*N* = 6,486); Geography (*N* = 3,181); SGM (*N* = 1,980); and Education (*N* = 279)

Utilizing participant-provided survey responses and registration information, participants were identified by the *All of Us* DRC as belonging to none, one, or multiple program UBR categories. As detailed in Fig. 1, 92.6% of VA-enrolled Veteran participants were identified as belonging to at least one UBR category. Interestingly, 33.4% of VA-enrolled Veteran participants were identified as belonging to two UBR categories, and 33.2% met the criteria for at least three UBR categories (Fig. 1), highlighting the importance of taking intersectionality into consideration. The highest percentages for individual categories (Fig. 2) were in age (58.3%), disability (47.1%), race/ethnicity (29.4%), income (27.4%), HAU (23.9%) and geography (11.7%), with lower frequencies for SGM (7.3%) and education (1.0%).

**Fig. 3:**
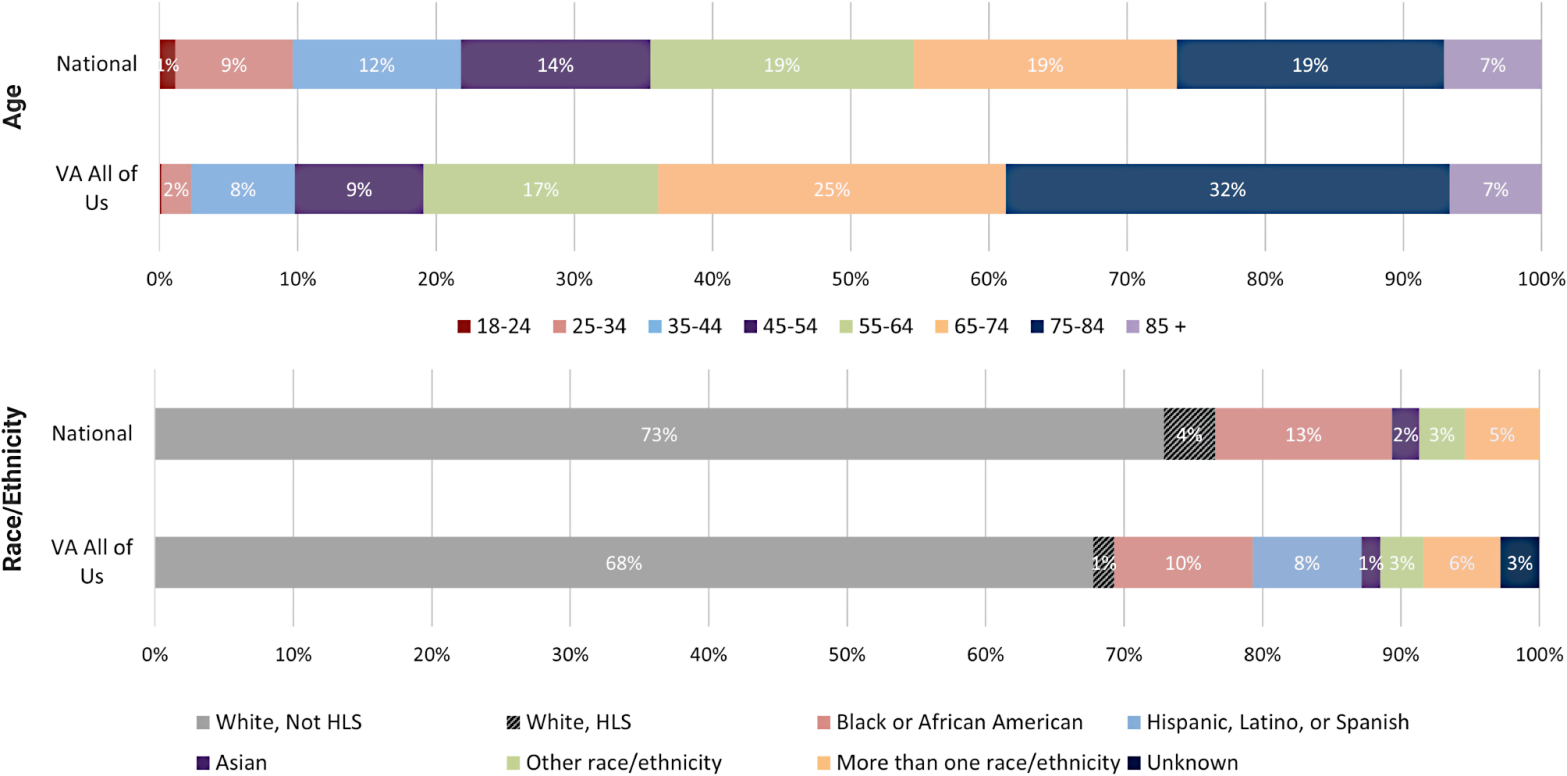
Veteran Age and Race/Ethnicity Characteristics. Demographic data are represented by percentages for age ranges in years and race/ethnicity. HLS = Hispanic, Latino, or Spanish. Data represent living U.S. Veterans nationally (*N* = 18,266,970) and Veterans enrolled in *All of Us* through the VA (*N* = 27,083)

Of the approximately 18 million living Veterans in 2023, data from the National Center for Veterans Analysis and Statistics indicated that approximately 27.1% were considered non-White Veterans, 64.5% were 55 years or older, and 88.6% of Veterans were male and 11.4% female [28]. Fig. 3 shows that while Veteran participants enrolled in *All of Us* are reflective of the overall national Veteran population, we were able to capture a slightly higher representation of non-White Veterans (29.4% as compared to 27.1%) and Veterans over the age of 65 (64.0% as compared to 45.5%). Note that HLS is reported differently across the *All of Us* and national data sources, allowing us to identify Veterans who consider HLS as their sole racial/ethnic identity in the *All of Us* data but not the national data.

The demographic complexity of Veteran research participants is demonstrated in Fig. 2, displaying UBR categories individually and in any represented combination. Notably, well over 75 unique UBR combinations were identified based on the eight UBR categories reported. Interestingly, the most prominent intersecting UBR categories were age and disability (29.4%), age and income (15.1%), race/ethnicity and disability (14.7%), and income and disability (14.6%). Other sizable participant UBR overlap included healthcare access and utilization (HAU) and disability (12.3%), race/ethnicity and age (12.1%), HAU and age (10.5%), race/ethnicity and income (10.5%), and HAU and income (9.8%). Veteran participants who met the criteria for the aforementioned intersections often met the criteria for other UBR categories as well; for example, of the 29.4% of participants who were UBR by both age and disability, 59.0% met the criteria for at least one other UBR category. Notably, 92.0% of VA participants who were UBR by geography also fell into one or more additional UBR categories, with 29.4% falling into three or more additional categories, suggesting that rural-living Veterans may have some of the most complex identities.

## Discussion

Diversity and inclusion in biomedical research are necessary to ensure advances in healthcare can be tailored to meet individual needs. U.S. Military Veterans are well established as an underrepresented group with unique exposures and health experiences of key importance to developing and implementing healthcare advances. Recent studies conducted at the VA have demonstrated that targeted recruitment strategies can promote Veteran representativeness in research [32, 33]. However, developing tailored approaches to drive Veteran representation requires an examination and understanding of Veteran diversity and identity focused on characteristics beyond age, sex, and race/ethnicity descriptors. Alongside these often-used measures of diversity, geographic differences and lived experience in health and wellness are of growing importance to research, particularly in combination with each other. Here we present evidence supporting previously undescribed Veteran intersectionality including 75 unique UBR combinations, and highlight the value of employing community-tailored methods and practices to better engage diverse participants in health research.

VA recruited and enrolled Veterans broadly for *All of Us* since 2018. Initial comparison of national Veteran demographics (age, sex, race/ethnicity) show our enrolled Veterans to be reflective of the U.S. Veteran population with a few differences (Fig. 3). Notable areas of difference between enrollee and national demographics are age and HLS identity. *All of Us* Veterans skew to those over the age of 65, likely due to their frequent use of VA facilities and availability during daytime clinic hours. Veterans identifying as HLS are noted in the *All of Us* data but not in the national demographics. *All of Us* allows participants to self-identify as HLS independent of other race/ethnicity categories. National Veteran demographics do not present HLS as a sole identity. We acknowledge differences in data collection and reporting methods for the two datasets and detail our efforts to make them comparable for the purposes of this analysis while still reporting key features unique to the *All of Us* dataset. While analysis of age, sex, and race/ethnicity demographics of VA *All of Us* participants detail a group that reflect the diversity of Veterans nationally, it is not necessarily a representative sample as the program is open to all Veterans [31].

Use of engagement approaches to meet distinct challenges (Table 1) allowed us to recruit and enroll a cohort of Veterans with complex identities (Figs. 1 and 2). For example, approximately one third of our participants met the criteria for three or more UBR categories (Fig. 1). Notable to us, 29.4% of those considered UBR by geography also met the criteria for three or more additional UBR categories, the most sizeable of which were age, disability, and income (Fig. 2), giving insight into often overlooked marginalization in more rural settings. While we have only piloted and conducted a small number of each type of engagement approach and therefore cannot report on comprehensive outcomes, the deployment of community-specific engagement and enrollment methods provided research opportunities to a broad segment of the Veteran population. Through our experience, we acknowledge that some engagement approaches appeared to be better received regionally and within specific groups than others, and have learned it is important to explore a variety of methods and seek regular community feedback to find what works best. Regardless of whether eventual enrollment stemmed from remote engagement, partnership with specific groups and community hubs, or our participant champions, to name a few examples, each of our engagement approaches took into account important factors (e.g., distance to an enrolling clinic, mobility, time, high-touch support, participant-to-participant referral, and trust building), which ultimately enabled us to spread program awareness in unique ways to greater and more diverse facets of the Veteran community.

Using *All of Us* operational data, our findings are the first to elucidate the complexity of Veteran identity beyond the scope of national statistics, with well over 75 unique UBR combinations identified among our participants. Though basic Veteran demographics are largely shaped by military recruitment and draft policies, shedding light on why many Veterans alive today identify as older, cisgender men [34], the broad range of datapoints (e.g., gender identity, access to healthcare, disability status, educational attainment, income, and geographical residence) collected by *All of Us* and viewed in combination demonstrates greater depth in demographics and health/wellness experiences than is often considered in enrollment reporting. Insights presented by Sabatello et al. [1] and Mapes et al. [6] suggest that identity and lived experience have a profound impact on health outcomes, lending weight to the need to consider intersectionality as a key variable in biomedical research.

The data presented here serve as a starting point for considering intersectional identity when planning and developing Veteran-specific programs. In the near future, ongoing changes to military recruitment strategy and policy will result in a more diverse Veteran landscape, including an increase in the *proportion* of Veterans from non-White racial and ethnic backgrounds (defined by VA as racial and ethnic minorities) from 27.5% in 2024 to 39.1% in 2053, women Veterans from 11.7% (2024) to 18.7% (2053), and younger Veterans (under the age of 45) from 21.9% (2024) to 25.5% (2053) [28], as well as growth in transgender and gender diverse-identifying Veterans receiving care at the VA [35]. Taken together, projections of Veteran population shifts, and the increasing importance of multi-faceted identity and lived experience for health outcomes, stress the importance of examining and understanding Veteran intersectionality. Considering complex and changing intersectional identities can ensure purposeful approaches to research representation and development of policy and practice to meet changing healthcare needs.

In response to shifts in Veteran identity over the years, VA has made great strides to meet the needs of specific communities, including creation of the Office of Rural Health in 2007, which works to reduce barriers that rural-living Veterans experience in receiving healthcare services [36], implementing the PACT Act passed in 2022, which expands eligibility for VA services to Veterans exposed to toxins during their military service [37], and the establishment of the Inclusion, Diversity, Equity, and Access Council in 2023 [38] and the Office of Health Equity LGBT Workgroup [39], both intended to identify inequities and offer solutions to delivery of benefits and services. VA efforts also include increased healthcare services offered to women Veterans and assignment of a Women Veterans Program Manager at every VA medical center [40]. We partnered with several of these groups to create community-based approaches to engage Veterans in the *All of Us* Research Program.

Our effort to engage and enroll a diverse group of Veterans in the *All of Us* Research Program was not without limitations. Funding constraints and protocol requirements limited our options for engagement, leading us to limit the frequency of outreach requiring staff travel, and sometimes seek out less costly solutions. Accessibility to digital devices and broadband internet required for *All of Us* enrollment also presented challenges, leaving some Veterans unable to complete program registration and enrollment without in-person support at an enrollment clinic. We also encountered Veterans who did not have access to transportation to a participating clinic and/or lived too far away but required in-person support, limiting our ability to assist them. Trust proved to be another obstacle to participation, underlining the importance of providing transparency throughout the research process, working to alleviate privacy concerns, and establishing localized personal relationships between program staff and participants.

While ours is just one research study and accounts for a small percentage of the Veteran population, Veterans are an important representation of the larger U.S. population and greater understanding of their intersectional identities is necessary for development of equitable and inclusive policies and programs that will better serve them [17]. In particular, more rigorous engagement of wide-ranging Veteran communities and groups - going beyond standard diversity considerations like age, sex, and race/ethnicity for research participation - is necessary to accurately identify their unique perspectives and needs. We have shared approaches to broaden participation aimed at increasing inclusion and present an intersectional view of the diversity of Veterans, but significant work is still needed to bring the concept of multi-faceted identity and the needs of the multiply marginalized to the forefront of planning for population-focused research. Ongoing program efforts are focused on further engaging women, rural-living, and sexual and gender minority identifying Veterans to better understand additional meaningful facets in their complex identities aiming to ensure inclusion in research.

## Conclusions

Accounting for proportional shifts in the Veteran landscape in years to come will be instrumental in developing research methodologies and healthcare goals that ensure Veteran experiences and needs are broadly accounted for. Data presented here take the first step in understanding Veteran identity by applying an intersectionality lens and denoting over 75 unique intersections among UBR categories for a segment of Veterans enrolled in the *All of Us* Research Program. Future endeavors to engage Veterans in research and serve Veteran care needs will benefit from continued exploration of engagement approaches cognizant of complex and intersecting identities.

## Data Availability

*All of Us* Research Program data is broadly available via the *All of Us* Research Program Researcher Workbench to registered users.

## Abbreviations

UBR: Underrepresented in biomedical research
SES: Socioeconomic status
Veterans: United States Military Veterans
All of Us: *All of Us* Research Program
NIH: National Institutes of Health
VA: Department of Veterans Affairs
EHR: Electronic health record
CBOC: Community-based outpatient clinic
BYOK: Bring your own kit
DRC: (All of Us) Data and Research Center
SGM: Sexual and gender minority
HAU: Healthcare access and utilization
HLS: Hispanic, Latino, or Spanish

## References

[1] Sabatello M, Diggs-Yang G, Santiago A, Easter C, Jacoby Morris K, Hollister BM, et al. The need for an intersectionality framework in precision medicine research. Am J Hum Genet. 2023;110(10):1609–15. 37802041 10.1016/j.ajhg.2023.08.013 PMC10577071

[2] Brown KE, Fohner AE, Woodahl EL. Beyond the individual: Community-centric approaches to increase diversity in biomedical research. Clin Pharmacol Ther. 2022;113(3):509–17. 36448873 10.1002/cpt.2808 PMC12162141

[3] Popejoy AB, Fullerton SM. Genomics is failing on diversity. Nature. 2016;538(7624):161–4. 27734877 10.1038/538161a PMC5089703

[4] Hamel LM, Penner LA, Albrecht TL, Heath E, Gwede CK, Eggly S. Barriers to clinical trial enrollment in racial and ethnic minority patients with cancer. Cancer Control. 2016;23(4):327–37. 27842322 10.1177/107327481602300404 PMC5131730

[5] Routen A, Bodicoat D, Willis A, Treweek S, Paget S, Khunti K. Tackling the lack of diversity in health research. Br J Gen Pract. 2022;72(722):444–7.

[6] Mapes BM, Foster CS, Kusnoor SV, Epelbaum MI, AuYoung M, Jenkins G, et al. Diversity and inclusion for the All of Us Research Program: A scoping review. PLoS ONE. 2020;15(7):e0234962. 32609747 10.1371/journal.pone.0234962 PMC7329113

[7] Baciu A, Negussie Y, Geller A, Weinstein JN, editors. Communities in action: pathways to health equity. Washington (DC): National Academies Press (US); 2017. 28418632

[8] Radley DC, Shah A, Collins SR, Powe NR, Zephyrin LC. Advancing Racial Equity in U.S. Health Care: The Commonwealth Fund 2024 State Health Disparities Report (Commonwealth Fund, 2024). 10.26099/vw02-fa96

[9] Ellingrud K, Pérez L, Petersen A, Sartori V, editors. Closing the Women’s Health Gap: A $1 Trillion Opportunity to Improve Lives and Economies. World Economic Forum; 2024.

[10] Campbell GM, Perry MP, Milford J, Murphy D. Personalising veteran healthcare: recognising barriers to access for minority and under-represented groups of veterans. BMJ Mil Health. 2024;170(5):446–50. 38897640 10.1136/military-2024-002768

[11] Hindorff LA, Bonham VL, Brody LC, Ginoza MEC, Hutter CM, Manolio TA, Green ED. Prioritizing diversity in human genomics research. Nat Rev Genet. 2018;19(3):175–85. 29151588 10.1038/nrg.2017.89 PMC6532668

[12] Velicer WF, Redding CA, Sun X, Prochaska JO. Demographic variables, smoking variables, and outcome across five studies. Health Psychol. 2007;26(3):278–87. 17500614 10.1037/0278-6133.26.3.278

[13] Homan P, Brown TH, King B. Structural intersectionality as a new direction for health disparities research. J Health Soc Behav. 2021;62(3):350–70. 34355603 10.1177/00221465211032947 PMC8628816

[14] Matthews LJ, Martschenko DO, Sabatello M. The value of intersectionality for genomic research on human behavior. Genet Med. 2023;25(7):100860. 37092536 10.1016/j.gim.2023.100860 PMC10330207

[15] Eckstrand KL, Eliason J, St Cloud T, Potter J. The priority of intersectionality in academic medicine. Acad Med. 2016;91(7):904–7. 27166867 10.1097/ACM.0000000000001231

[16] Crenshaw K. Demarginalizing the intersection of race and sex: A black feminist critique of antidiscrimination doctrine, feminist theory and antiracist politics. Univ Chic Legal Forum. 1989;1989(1):139–68.

[17] Blevins KR, Blevins AL. Advocating for minority veterans in the United States: principles for equitable public policy. J Military Veteran Family Health. 2021;7(s1):136–42.

[18] Knelson LP, Cukras AR, Savoie J, Agarwal A, Guo H, Hu J, et al. Barriers to clinical trial accrual: perspectives of community-based providers. Clin Breast Cancer. 2020;20(5):395–e4013. 32605813 10.1016/j.clbc.2020.05.001

[19] Leira EC, Viscoli CM, Polgreen LA, Gorman M, Kernan WN. Distance from home to research center: A barrier to in-person visits but not treatment adherence in a stroke trial. Neuroepidemiology. 2018;50(3–4):137–43. 29587267 10.1159/000486315 PMC5975097

[20] Denny JC, Rutter JL, Goldstein DB, Philippakis A, Smoller JW, Jenkins G, et al. The All of Us Research Program. N Engl J Med. 2019;381(7):668–76. 31412182 10.1056/NEJMsr1809937 PMC8291101

[21] National Institute on Minority Health and Health Disparities, History L. https://www.nimhd.nih.gov/about/legislative-info/legislative-history.html. Accessed 31 March 2025.

[22] All of Us Research Hub. https://www.researchallofus.org. Accessed 4 Janaury 2025.

[23] Ramirez AH, Sulieman L, Schlueter DJ, Halvorson A, Qian J, Ratsimbazafy F, et al. The All of Us Research Program: data quality, utility, and diversity. Patterns (NY). 2022;3(8):100570. 10.1016/j.patter.2022.100570 PMC9403360 36033590

[24] Smith LH, Wang W, Keefe-Oates B. Pregnancy episodes in All of Us: Harnessing multi-source data for pregnancy-related research. J Am Med Inf Assoc. 2024;31(12):2789–99. 10.1093/jamia/ocae195 PMC11631125 39043412

[25] Kim BY, Anthopolos R, Do H, Zhong J. Model-based estimation of individual-level social determinants of health and its applications in All of Us. J Am Med Inf Assoc. 2024;31(12):2880–9. 10.1093/jamia/ocae168 PMC11631124 39003521

[26] Barker AM, Dunlap S, Hartmann CW, Wilson-Menzfeld G, McGill G. Engaging veterans in the research process: a practical guide. J Comp Eff Res. 2022;11(10):751–64. 35699110 10.2217/cer-2022-0010

[27] Olenick M, Flowers M, Diaz VJ. US veterans and their unique issues: enhancing health care professional awareness. Adv Med Educ Pract. 2015;6:635–9. 26664252 10.2147/AMEP.S89479 PMC4671760

[28] National Center for Veterans Analysis and Statistics, Veteran Population. https://www.va.gov/vetdata/veteran_population.asp. Accessed 16 January 2025.

[29] Sharma Y, Saha A, Goldsack JC. Defining the dimensions of diversity to promote inclusion in the digital era of health care: A lexicon. JMIR Public Health Surveill. 2024;10:e51980. 38335013 10.2196/51980 PMC10891484

[30] Million Veteran Program. Researcher Hub: MVP Data Overview. https://www.mvp.va.gov/pwa/discover-mvp-data. Accessed 31 March 2025.

[31] All of Us Research Hub Frequently Asked Questions. https://www.researchallofus.org/faq. Accessed 4 January 2025.

[32] Vassy JL, Brunette CA, Lebo MS, MacIsaac K, Yi T, Danowski ME, et al. The GenoVA study: equitable implementation of a pragmatic randomized trial of polygenic-risk scoring in primary care. Am J Hum Genet. 2023;110(11):1841–52. 37922883 10.1016/j.ajhg.2023.10.001 PMC10645559

[33] Whitbourne SB, Li Y, Brewer JVV, Deen J, Gutierrez C, Murphy SA, et al. Overview of efforts to increase women enrollment in the Veterans Affairs Million Veteran Program. Health Equity. 2023;7(1):324–32. 37284530 10.1089/heq.2023.0006 PMC10240313

[34] Porter B. On the utility of using the All of Us Research Program as a resource to study military service members and veterans. J Am Med Inf Assoc. 2024;31(12):2958–61. 10.1093/jamia/ocae153 PMC11631084 38894629

[35] Wolfe HL, Boyer TL, Rodriguez KL, Klima GJ, Shipherd JC, Kauth MR, Blosnich JR. Exploring research engagement and priorities of transgender and gender diverse veterans. Military Med. 2023;188(5–6):e1224–31. 10.1093/milmed/usab460 34791410

[36] Office of Research and Development, Rural Health. https://www.research.va.gov/topics/rural_health.cfm. Accessed 16 January 2025.

[37] The PACT Act and your VA benefits. Accessed 31 March 2025. https://www.va.gov/resources/the-pact-act-and-your-va-benefits

[38] VA stands up Agency Equity Team to ensure that all Veterans receive the world-class care and benefits they deserve [press release]. June 23, 2023.

[39] Mattocks KM, Kauth MR, Sandfort T, Matza AR, Sullivan JC, Shipherd JC. Understanding health-care needs of sexual and gender minority veterans: how targeted research and policy can improve health. LGBT Health. 2014;1(1):50–7. 26789509 10.1089/lgbt.2013.0003

[40] More than 50,000 women Veterans enrolled in VA health care over past 365 days, marking the largest enrollment year ever for women Veterans [press release]. June 12, 2024.

